# Association of serum Sema4D and ANGPTL4 levels with disease severity and prognosis in myasthenia gravis patients

**DOI:** 10.3389/fneur.2026.1965303

**Published:** 2026-10-26

**Authors:** Wenqian Li, Mengsi Zhang, Zhibo Wang, Chundong Gong, Lu Sun, Wenjing Feng, Guoyan Qi, Ying Yang, Fuda Yu

**Affiliations:** 1 Department of Neuroelectrophysiology, Shijiazhuang People’s Hospital, Shijiazhuang, China; 2 Center for Myasthenia Gravis, Shijiazhuang People’s Hospital, Shijiazhuang, China

**Keywords:** angiopoietin-like protein 4, disease severity, myasthenia gravis, prognosis, semaphorin 4D

## Abstract

**Objective:**

To investigate the relationship of serum semaphorin 4D (Sema4D) and angiopoietin-like protein 4 (ANGPTL4) levels with disease severity and prognosis in anti–acetylcholine receptor (AChR) antibody–positive myasthenia gravis (MG) patients.

**Methods:**

Between December 2023 and April 2025, 468 anti-AChR antibody–positive MG patients hospitalized at our center were prospectively enrolled and divided into good- and poor-prognosis groups according to whether they achieved minimal manifestations (MM) or better status at the 1-year follow-up. Healthy controls were also enrolled. Serum Sema4D and ANGPTL4 levels were measured by enzyme-linked immunosorbent assay (ELISA). Correlations were evaluated by Spearman’s rank correlation. Multivariable logistic regression was used to identify independent risk factors for poor prognosis. Receiver operating characteristic (ROC) curve analysis was used to evaluate the predictive value of the two biomarkers alone and in combination, and clinical and combined models were developed to evaluate the biomarkers’ incremental predictive value.

**Results:**

Serum Sema4D and ANGPTL4 levels were significantly higher in MG patients than in healthy controls (both *p* < 0.001). Compared with the good-prognosis group, the poor-prognosis group had higher proportions of Myasthenia Gravis Foundation of America (MGFA) class IV-V, dysphagia, and thymoma, and higher Myasthenia Gravis Quality of Life 15-item revised (MG-QoL15r) and Controlling Nutritional Status (CONUT) scores (all *p* < 0.05), and significantly higher levels of both biomarkers (both *p* < 0.05). Both Sema4D and ANGPTL4 correlated positively with MG-QoL15r and CONUT scores and with each other (*r* = 0.431, *p* < 0.001). Multivariable logistic regression showed that MGFA class IV-V, dysphagia, thymoma, higher MG-QoL15r and CONUT scores, and elevated serum Sema4D and ANGPTL4 levels were all independent risk factors for poor prognosis (all *p* < 0.05). The combination of the two biomarkers yielded an area under the ROC curve (AUC) of 0.841, superior to either marker alone. Adding the biomarkers to the clinical model significantly improved prognostic discrimination, with the AUC increasing from 0.874 to 0.907 (*p* = 0.0002).

**Conclusion:**

Combined measurement of serum Sema4D and ANGPTL4 is associated with disease severity and poor prognosis in anti-AChR antibody–positive MG and shows good predictive value for patient prognosis.

## 1 Introduction

Myasthenia gravis (MG) is an antibody-mediated autoimmune disease of the neuromuscular junction (NMJ), in which autoantibodies against the acetylcholine receptor (AChR) and other postsynaptic membrane-associated proteins impair neuromuscular transmission. Clinically, it is characterized by fluctuating skeletal muscle weakness and fatigability; in severe cases, respiratory muscle involvement may progress to life-threatening myasthenic crisis (1, 2). In recent years, the prevalence of MG has continued to rise with population aging, advances in diagnostic techniques, and improved diagnostic capabilities. A global systematic review published in 2025 reported a median prevalence of approximately 129.6 per million population, with marked regional variation—higher in developed Western countries and lower in Asian countries (3). Although treatments including corticosteroids, immunosuppressants, intravenous immunoglobulin, and targeted biologics have significantly improved survival and quality of life, MG remains a clinically heterogeneous disease, with considerable interindividual variation in age at onset, affected muscle groups, disease severity, treatment response, and long-term prognosis (4). Studies have shown that some patients fail to achieve sustained remission despite standard treatment, and adverse outcomes such as repeated relapses, disease progression, and myasthenic crisis remain common, imposing a heavy functional and socioeconomic burden on patients (5, 6). Therefore, identifying biomarkers closely associated with MG disease activity, severity, and prognosis is of great clinical significance for improving risk stratification, optimizing individualized treatment strategies, and enhancing long-term outcomes.

In addition to classical autoantibody-mediated injury, aberrant immune cell activation, cytokine imbalance, and altered vascular endothelial function have also been implicated in the progression of MG (7). Semaphorin 4D (Sema4D) is a transmembrane glycoprotein widely expressed on various immune cells; by binding to its receptors, it regulates lymphocyte activation and inflammatory responses (8). Sema4D is aberrantly expressed in autoimmune diseases such as rheumatoid arthritis and multiple sclerosis, and is regarded as an important mediator linking immune responses to tissue damage (9, 10). Angiopoietin-like protein 4 (ANGPTL4), a member of the angiopoietin-like protein family, not only participates in lipid metabolism but also contributes to immune-inflammatory responses by modulating inflammatory cell recruitment and tissue microenvironment remodeling; its dysregulated expression is closely associated with various inflammatory and neurological disorders (11). In rheumatoid arthritis, ANGPTL4 promotes bone destruction, whereas in multiple sclerosis, its elevated expression impairs myelin clearance and thereby inhibits repair—suggesting that ANGPTL4 may contribute to disease progression in autoimmune diseases by promoting inflammation and tissue damage (12, 13). However, studies on the expression of Sema4D and ANGPTL4 in MG and their relationships with disease severity, clinical classification, and prognosis remain very limited, and evidence simultaneously evaluating the clinical value of both markers is particularly lacking.

Therefore, this study measured serum Sema4D and ANGPTL4 levels in patients with MG and investigated their associations with disease severity and prognosis, aiming to provide novel biomarkers and a theoretical basis for disease assessment and prognostic prediction in MG.

## 2 Materials and methods

### 2.1 Study population

Based on prior literature, the proportion of MG patients who failed to achieve minimal manifestation (MM) or better status at 1 year after immunotherapy was approximately 19% (14). The minimum required sample size was calculated to be 255 using PASS 15.0 software, with an absolute precision of 5% (i.e., a 10% confidence interval width) and a two-sided significance level of 0.05. To explore relevant risk factors, binary logistic regression analysis was planned. According to the events per variable (EPV) principle for predictive model construction, the inclusion of 7 candidate predictor variables required at least 70 adverse outcome events; given an event rate of 19%, 368 patients were required to complete follow-up. After further accounting for a 10% loss to follow-up, no fewer than 410 patients were required at the start of follow-up. Accordingly, 468 MG patients were initially enrolled.

*Inclusion criteria*: (1) MG patients who were seropositive for AChR antibodies (15), including those with pure ocular myasthenia gravis (Myasthenia Gravis Foundation of America [MGFA] class I) and generalized myasthenia gravis (MGFA class II–V); (2) age 18–75 years; (3) scheduled to receive immunosuppressive therapy; and (4) written informed consent obtained from the patients or their family members.

*Exclusion criteria*: (1) coexisting autoimmune disorders (e.g., systemic lupus erythematosus and rheumatoid arthritis); (2) concomitant malignancies, except thymoma; (3) active infection; (4) severe hepatic or renal insufficiency; (5) recent use of immunosuppressants or biologic agents, with the exception of cholinesterase inhibitors such as pyridostigmine used for symptomatic treatment; (6) pregnancy or lactation; and (7) incomplete clinical data.

This was a prospective cohort study. A total of 468 MG patients who presented to our hospital from December 2023 to April 2025 were enrolled. Among them, 192 (41.03%) were newly diagnosed, whereas 276 (58.97%) had been previously diagnosed at other hospitals and referred to our center. Fifty-three patients were excluded according to the inclusion and exclusion criteria, and 25 were lost to follow-up during the study period. Ultimately, 390 MG patients were included in the final analysis. None of these patients had received immunosuppressive therapy at the time of enrollment. Although some patients had a long disease duration, they had not previously received immunosuppressive therapy, mainly for the following reasons: (1) the diagnosis had not been established previously, or the diagnosis had only been suspected but not confirmed at other hospitals, and treatment had therefore not been standardized; (2) although the diagnosis had been established, the symptoms were mild in the early stage of the disease, and a watch-and-wait strategy had been adopted; and (3) treatment had been delayed due to patient-related factors. Among the patients previously managed with a watch-and-wait strategy, immunosuppressive therapy was initiated because of symptom worsening or progression from ocular to generalized disease; these patients therefore met the inclusion criterion of being scheduled to receive immunosuppressive therapy at enrollment. Some of these patients had received symptomatic treatment with pyridostigmine before enrollment. Pyridostigmine was not required to be discontinued before blood sampling; however, medication use was recorded and analyzed in the between-group comparisons. During the same period, healthy individuals undergoing routine physical examinations were enrolled as the control group, frequency-matched by age and sex to ensure comparability between the two groups. The inclusion criteria for healthy controls were as follows: (1) age 18–75 years; (2) no history of autoimmune diseases, neurological diseases, or malignancies; (3) no acute or chronic infections; (4) normal hepatic and renal function; and (5) no use of immunosuppressants or glucocorticoids within the preceding 3 months. The exclusion criteria for healthy controls were as follows: (1) abnormal findings on physical examination requiring further evaluation and management; (2) pregnancy or lactation; and (3) refusal to participate. Following the 1-year follow-up period, all MG patients were categorized into the good-prognosis group and the poor-prognosis group according to the MGFA post-intervention status classification. The study participant flowchart is shown in Figure 1.

**Figure 1:**
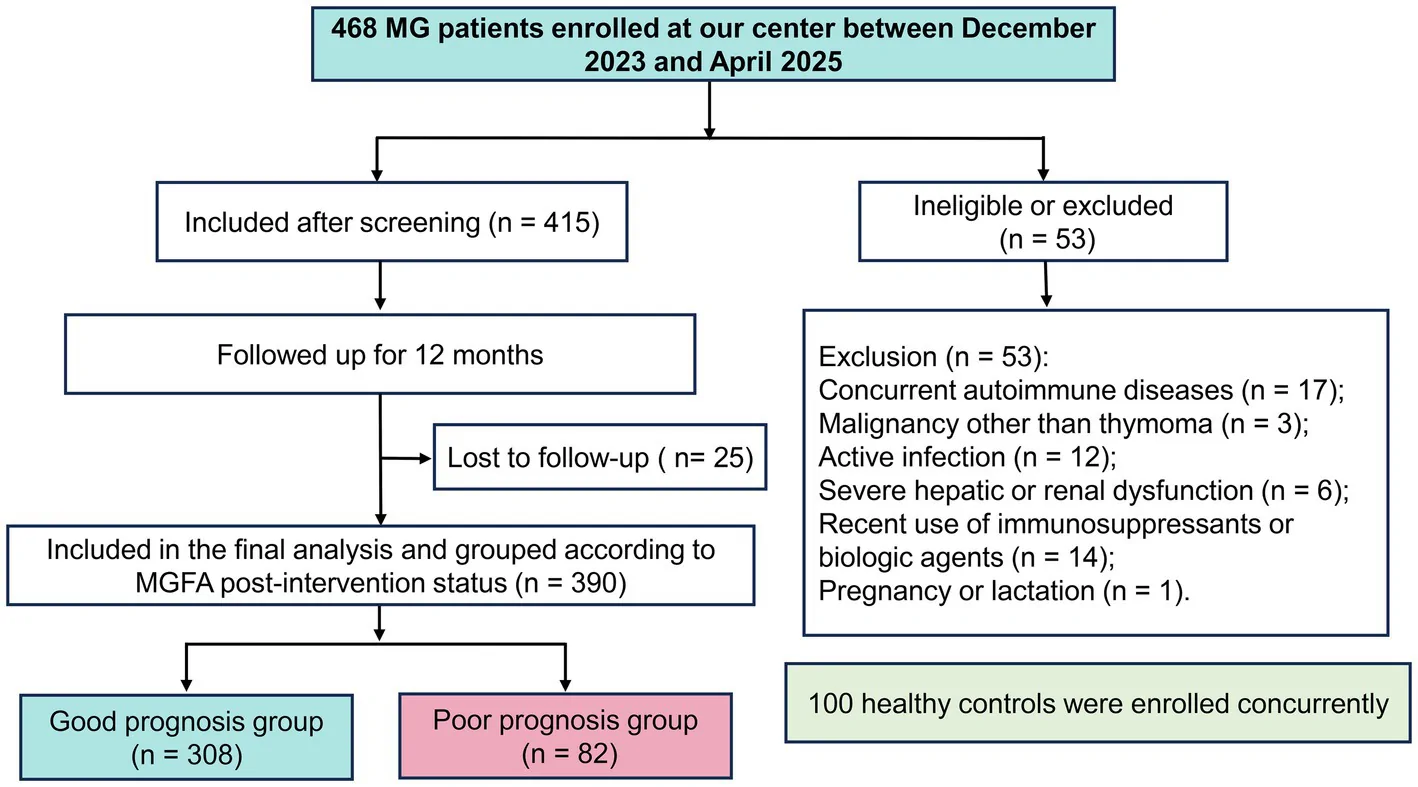
Study participant flowchart.

### 2.2 Clinical data collection

The following clinical data were collected from patients with MG: age, sex, body mass index (BMI), hypertension, diabetes mellitus, disease duration, pyridostigmine use before enrollment, MGFA clinical classification, age at onset, baseline dysphagia, thymoma, Myasthenia Gravis Quality of Life 15-item revised (MG-QoL15r) score, Controlling Nutritional Status (CONUT) score, serum total protein (TP), and C-reactive protein (CRP). Disease duration was defined as the interval from the onset of the first symptom to blood sampling at enrollment. The MGFA clinical classification was assessed at enrollment (before serum sampling) according to the Foundation’s criteria and categorized into classes I–V (15). Patients were categorized according to age at onset as early-onset (≤50 years) or late-onset (>50 years). Quality of life was assessed using the MG-QoL15r, which yields a total score ranging from 0 to 60, with higher scores indicating poorer quality of life and greater disease burden (16). Nutritional status was assessed using the CONUT score, which is calculated from serum albumin, total lymphocyte count, and total cholesterol and ranges from 0 to 12, with higher scores indicating more severe malnutrition (17). Bulbar muscle involvement in patients with MG may lead to inadequate dietary intake, and malnutrition is common in this population and associated with poor prognosis. As the CONUT score is based on objective biochemical parameters, it avoids subjective bias and has been widely applied in prognostic assessment of chronic diseases; it was therefore included in this study to investigate its independent association with prognosis in MG. The Myasthenia Gravis Activities of Daily Living (MG-ADL), Quantitative Myasthenia Gravis (QMG), and Myasthenia Gravis Composite (MGC) scores were not systematically collected as routine follow-up measures owing to limited outpatient follow-up time and assessment resources.

### 2.3 Serum Sema4D and ANGPTL4 measurement

Peripheral venous blood was collected from all enrolled MG patients before treatment. For newly diagnosed patients, blood sampling was performed after confirmation of the diagnosis and before the initiation of any treatment; for referred patients with an established diagnosis, sampling was performed after transfer to our hospital for evaluation and before the formulation or initiation of an immunosuppressive regimen. For healthy controls, 5 mL of fasting peripheral venous blood was collected on the morning of the physical examination day. After standing at room temperature for 2 h, the samples were centrifuged at 2500×g for 20 min, and the supernatant was collected. Serum levels of Sema4D and ANGPTL4 were measured by enzyme-linked immunosorbent assay (ELISA) using a Sema4D ELISA kit (cat. no. HM11208; Bioswamp, Wuhan, China) and an ANGPTL4 ELISA kit (cat. no. HM11906; Bioswamp, Wuhan, China), respectively, according to the manufacturer’s protocols. The optical density (OD) at 450 nm was read using a microplate reader (iMark, Bio-Rad, United States). All samples were tested in duplicate, and the intra- and inter-assay coefficients of variation (CVs) were both <10%; mean values were used for analysis. The entire assay procedure was performed by laboratory technicians blinded to the clinical data. Thus, serum sampling in all patients was completed before the initiation of any immunosuppressive therapy, ensuring that the measured biomarker levels reflected the untreated disease state.

### 2.4 Follow-up and prognostic evaluation criteria

All enrolled patients received standardized immunotherapy based on the 2020 international consensus guidance (18), including corticosteroids, immunosuppressants such as azathioprine or mycophenolate mofetil, and acute interventions such as intravenous immunoglobulin (IVIg) or plasma exchange for myasthenic crisis. Medication regimens followed standard clinical practice as recommended by the guidelines and were administered by attending physicians within a unified treatment framework. This study was an observational prognostic study and did not participate in treatment decisions.

Follow-up was performed at 3, 6, and 12 months after treatment, via outpatient clinic visits, telephone interviews, and review of the hospital medical record system. At 12 months, outcome assessment was performed by two attending neurologists who were blinded to the baseline serum biomarker results, according to the Myasthenia Gravis Foundation of America Post-Intervention Status (MGFA-PIS) criteria (15). The PIS categories included Complete Stable Remission (CSR), Pharmacologic Remission (PR), MM, Improved (I), Unchanged (U), Worse (W), and Death (D). The follow-up endpoint was 12 months after enrollment or the occurrence of an adverse prognostic event (myasthenic crisis or death).

Good prognosis was defined as achieving a PIS of Minimal Manifestations or better at the 1-year follow-up, encompassing Complete Stable Remission, Pharmacologic Remission, and Minimal Manifestations. Minimal Manifestations denoted the absence of functional symptoms attributable to myasthenia gravis, with certain muscle weakness remaining detectable on physical examination. This definition was consistent with the treatment goal recommended by the 2020 international consensus guidance (18, 19). Poor prognosis was defined as failure to achieve the above criteria at the 1-year follow-up.

### 2.5 Statistical analysis

All statistical analyses were performed using SPSS 26.0 and R software (version 4.3.1). Normally distributed continuous variables were expressed as mean ± standard deviation (SD), and between-group comparisons were performed using the t test. Non-normally distributed variables were expressed as median (Q1, Q3), and were compared using the Mann–Whitney *U* test. Categorical variables were expressed as counts and percentages (%), and were compared by the chi-square (*χ*^2^) test. *p* < 0.05 was considered statistically significant. Spearman’s rank correlation analysis was used to evaluate the correlations of serum Sema4D and ANGPTL4 levels with MG-QoL15r and CONUT scores. Receiver operating characteristic (ROC) curves were constructed to assess the value of serum Sema4D and ANGPTL4, alone and in combination, for predicting poor prognosis in patients with MG, and the area under the curve (AUC) was calculated. Multivariable logistic regression analysis was performed to identify independent risk factors for poor prognosis in patients with MG. Given that MG treatment regimens are formulated according to disease severity and activity and are closely associated with MGFA clinical classification, they represent derived variables of disease severity rather than independent confounders; therefore, specific treatment regimens were not included as independent variables in the analysis.

## 3 Results

### 3.1 Comparison of general clinical characteristics and serum Sema4D and ANGPTL4 levels between MG patients and healthy controls

Compared with healthy controls, serum Sema4D and ANGPTL4 levels were significantly higher in MG patients (both *p* < 0.001), whereas no significant between-group differences were observed in age, sex, or BMI, suggesting that these general clinical characteristics were comparable between the two groups (Table 1).

**Table 1:** Comparison of general clinical characteristics and serum Sema4D and ANGPTL4 levels between MG patients and healthy controls.

Variable	Healthy controls (*n* = 100)	MG group (*n* = 390)	*t*/*Z*/*χ*^2^	*p*
Age (years, mean ± SD)	53.09 ± 7.37	52.73 ± 7.85	0.413^#^	0.680
Sex [*n* (%)]			0.387^*^	0.534
Male	46 (46.00)	193 (49.49)		
Female	54 (54.00)	197 (50.51)		
BMI (kg/m^2^, mean ± SD)	22.31 ± 1.85	22.70 ± 2.07	−1.737^#^	0.083
Sema4D [*M* (P25, P75), ng/mL]	33.52 (30.01, 38.68)	43.52 (36.12, 51.01)	−8.619^†^	<0.001
ANGPTL4 [*M* (P25, P75), ng/mL]	12.20 (9.58, 14.57)	17.15 (14.34, 20.62)	−10.151^†^	<0.001

^*^ indicates *χ*^2^ value; ^†^ indicates *Z*-value; ^#^ indicates *t*-value.

### 3.2 Comparison of baseline characteristics between MG patients with different prognoses

Compared with the good-prognosis group, the poor-prognosis group had a higher proportion of patients with MGFA class IV-V (the good-prognosis group included 50, 120, 83, 45, and 10 patients with MGFA classes I, II, III, IV, and V, respectively, whereas the poor-prognosis group included 6, 18, 15, 28, and 15 patients, respectively), as well as higher proportions of baseline dysphagia and concomitant thymoma; in addition, both the MG-QoL15r and CONUT scores were significantly higher in the poor-prognosis group. All of these differences were statistically significant (all *p* < 0.05). No significant differences were observed between the two groups in age, BMI, hypertension, diabetes mellitus, disease duration, pyridostigmine use before enrollment, age at onset, TP, or CRP (all *p* > 0.05). Among the 390 patients who completed follow-up, the distribution of MGFA-PIS at 12 months was as follows: CSR in 25 patients (6.41%), PR in 43 (11.03%), MM in 240 (61.54%), I in 53 (13.59%), U in 19 (4.87%), W in 8 (2.05%), and D in 2 (0.51%). The good-prognosis group (CSR + PR + MM) comprised 308 patients (79.0%), and the poor-prognosis group (I + U + W + D) comprised 82 patients (21.0%) (Table 2). The distribution of treatments during follow-up is shown in Table 3. No significant differences were found between the two groups in pyridostigmine use or the proportions of immunosuppressive regimens (all *p* > 0.05); however, the proportions of patients receiving IVIg, plasma exchange, and thymectomy were significantly higher in the poor-prognosis group (all *p* < 0.05).

**Table 2:** Comparison of baseline clinical characteristics between MG patients with different prognoses.

Variable	Good-prognosis group (*n* = 308)	Poor-prognosis group (*n* = 82)	*t*/*Z*/*χ*^2^	*p*
Age (years, mean ± SD)	52.37 ± 7.75	54.07 ± 8.11	−1.747^#^	0.081
Sex [*n* (%)]			1.207^*^	0.272
Male	148 (48.05)	45 (54.88)		
Female	160 (51.95)	37 (45.12)		
BMI (kg/m^2^, mean ± SD)	22.64 ± 2.11	22.92 ± 1.89	−1.087^#^	0.278
Hypertension [*n* (%)]	71 (23.05)	23 (28.05)	0.884^*^	0.347
Diabetes mellitus [*n* (%)]	40 (12.99)	14 (17.07)	0.906^*^	0.341
Disease duration [months, median (Q1, Q3)]	14 (12, 17)	15 (12, 17)	−1.189^†^	0.234
Pyridostigmine use before enrollment [*n* (%)]			0.889	0.346
Yes	249 (80.84)	70 (85.37)		
No	59 (19.16)	12 (14.63)		
MGFA class [*n* (%)]			41.164^*^	<0.001
I–III	253 (82.14)	39 (47.56)		
IV-V	55 (17.86)	43 (52.44)		
Age at onset [*n* (%)]			1.991^*^	0.158
Early-onset	120 (38.96)	25 (30.49)		
Late-onset	188 (61.04)	57 (69.51)		
Baseline dysphagia [*n* (%)]			11.613^*^	0.001
Yes	67 (21.75)	33 (40.24)		
No	241 (78.25)	49 (59.76)		
Thymoma [*n* (%)]			9.677^*^	0.002
Present	34 (11.04)	20 (24.39)		
Absent	274 (88.96)	62 (75.61)		
MGFA-PIS [*n* (%)]			—	—
CSR	25 (6.41)	—		
PR	43 (11.03)	—		
MM	240 (61.54)	—		
I	—	53 (13.59)		
U	—	19 (4.87)		
W	—	8 (2.05)		
D	—	2 (0.51)		
MG-QoL15r score [points, median (Q1, Q3)]	11 (9, 14)	15 (11, 17)	−6.397^†^	<0.001
CONUT score [points, median (Q1, Q3)]	3 (2, 4)	4 (3, 5)	−6.235^†^	<0.001
TP [g/L, median (Q1, Q3)]	67 (64, 71)	66 (62, 70)	−1.536^†^	0.125
CRP (mg/L, mean ± SD)	8.26 ± 2.14	8.61 ± 1.81	−1.341^#^	0.181

^*^ indicates *χ*^2^ value; ^†^ indicates *Z*-value; ^#^ indicates *t*-value. Disease duration was defined as the interval from symptom onset to blood sampling at enrollment.

**Table 3:** Details of treatment initiated during follow-up.

Variable	Good-prognosis group (*n* = 308)	Poor-prognosis group (*n* = 82)	*t*/*Z*/*χ*^2^	*p*
Pyridostigmine use [*n* (%)]			0.142	0.707
Yes	298 (96.75)	80 (97.56)		
No	10 (3.25)	2 (2.44)		
Immunosuppressive regimen [*n* (%)]			1.201	0.753
Corticosteroids alone	88 (28.57)	20 (24.39)		
Corticosteroids + azathioprine	115 (37.34)	29 (35.37)		
Corticosteroids + mycophenolate mofetil	78 (25.32)	25 (30.49)		
Other immunosuppressive regimens^*^	27 (8.77)	8 (9.76)		
Acute-phase intervention [*n* (%)]				
IVIg	18 (5.84)	14 (17.07)	10.841	0.001
Plasma exchange	6 (1.95)	9 (10.98)	14.271	<0.001
Thymectomy [*n* (%)]			10.057	0.002
Yes	36 (11.69)	21 (25.61)		
No	272 (88.31)	61 (74.39)		

*Other immunosuppressive regimens included tacrolimus, cyclosporine, and cyclophosphamide. Some patients received multiple regimens sequentially and were classified according to the main regimen used during follow-up.

### 3.3 Comparison of serum Sema4D and ANGPTL4 levels between the good-prognosis and poor-prognosis groups

Compared with the good-prognosis group, the poor-prognosis group exhibited significantly higher serum Sema4D and ANGPTL4 levels (both *p* < 0.001) (Figures 2A,B). As shown in Figure 2C, the scatter plot of individual patients demonstrated that those with poor prognosis were predominantly distributed in the region where both biomarkers were concurrently elevated: 33 of 82 patients (40.2%) in the poor-prognosis group and 23 of 308 patients (7.5%) in the good-prognosis group had serum Sema4D and ANGPTL4 levels above their respective optimal cutoffs (48.24 and 20.35 ng/mL, respectively).

**Figure 2:**
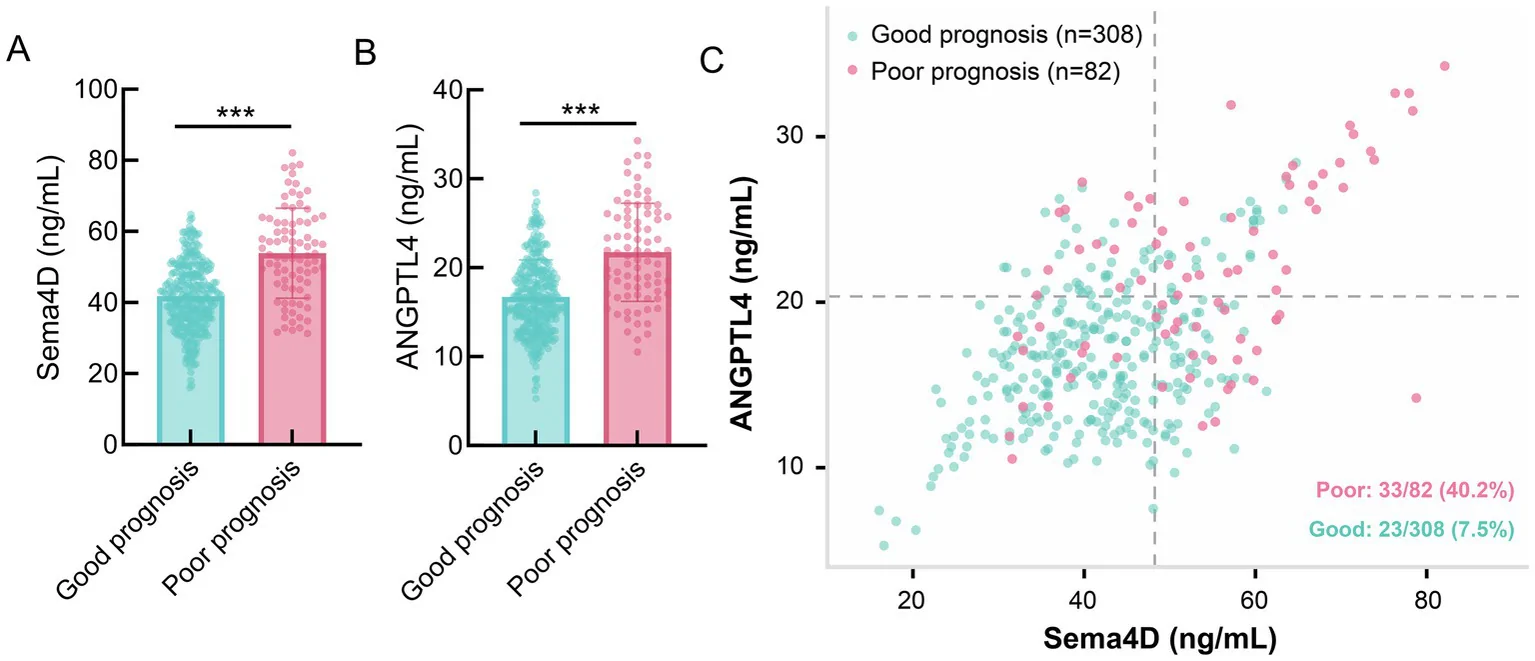
Comparison of serum Sema4D and ANGPTL4 levels between the good-prognosis and poor-prognosis groups. **(A)** Serum Sema4D levels; **(B)** serum ANGPTL4 levels; **(C)** scatter plot of serum Sema4D versus ANGPTL4 levels with individual patients color-coded by 1-year prognosis (teal, good-prognosis group; pink, poor-prognosis group). In **(C)**, the dashed lines indicate the optimal cutoffs for Sema4D (48.24 ng/mL) and ANGPTL4 (20.35 ng/mL), and the percentages in the lower-right denote the proportions of patients with concurrently elevated levels of both biomarkers. Individual data points are overlaid on the bars in **(A,B)** and color-coded by 1-year prognosis. ***p < 0.001 compared with the good-prognosis group.

### 3.4 Correlation of serum Sema4D and ANGPTL4 levels with MGFA clinical severity, quality of life, and nutritional status

The MGFA class used in this analysis were based on assessments performed at enrollment (baseline, before serum sampling). Serum Sema4D and ANGPTL4 levels were significantly higher in patients with MGFA class IV-V than in those with MGFA class I–III (both *p* < 0.001; Figures 3A,B). Serum Sema4D and ANGPTL4 levels were also significantly higher in the thymomatous MG (ThMG) group than in the non-thymomatous MG (Non-ThMG) group (both *p* < 0.001; Figures 3C,D). Spearman’s rank correlation analysis showed that Sema4D and ANGPTL4 levels were positively correlated with the MG-QoL15r score (quality-of-life burden) (Sema4D: *r* = 0.378, *p* < 0.001; ANGPTL4: *r* = 0.370, *p* < 0.001) and the CONUT score (nutritional status) (Sema4D: *r* = 0.355, *p* < 0.001; ANGPTL4: *r* = 0.369, *p* < 0.001; Figures 4A–D). In addition, serum Sema4D and ANGPTL4 levels were positively correlated with each other (*r* = 0.431, *p* < 0.001; Figure 4E).

**Figure 3:**
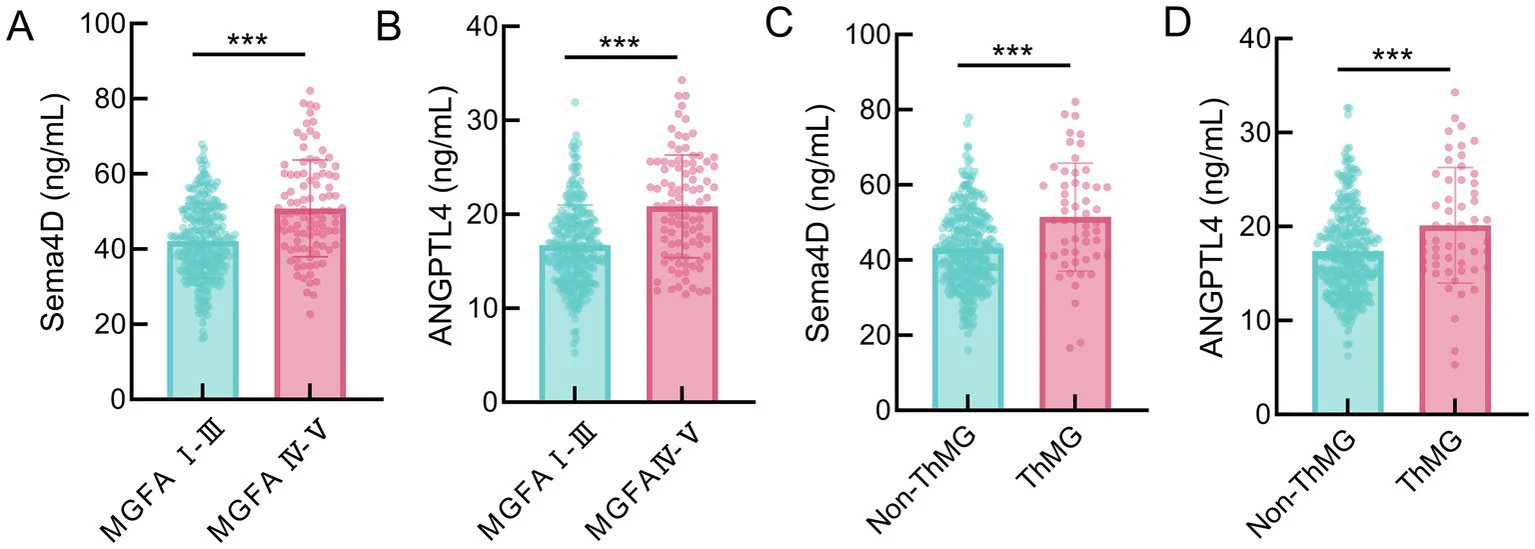
Comparison of serum Sema4D and ANGPTL4 levels among patients with different MGFA classes and thymoma status. **(A)** Serum Sema4D levels in patients with MGFA class I–III versus class IV-V; **(B)** serum ANGPTL4 levels in patients with MGFA class I–III versus class IV-V; **(C)** serum Sema4D levels in patients with versus without thymoma; **(D)** serum ANGPTL4 levels in patients with versus without thymoma. ***p < 0.001. ThMG, thymomatous myasthenia gravis; Non-ThMG, non-thymomatous myasthenia gravis.

**Figure 4:**
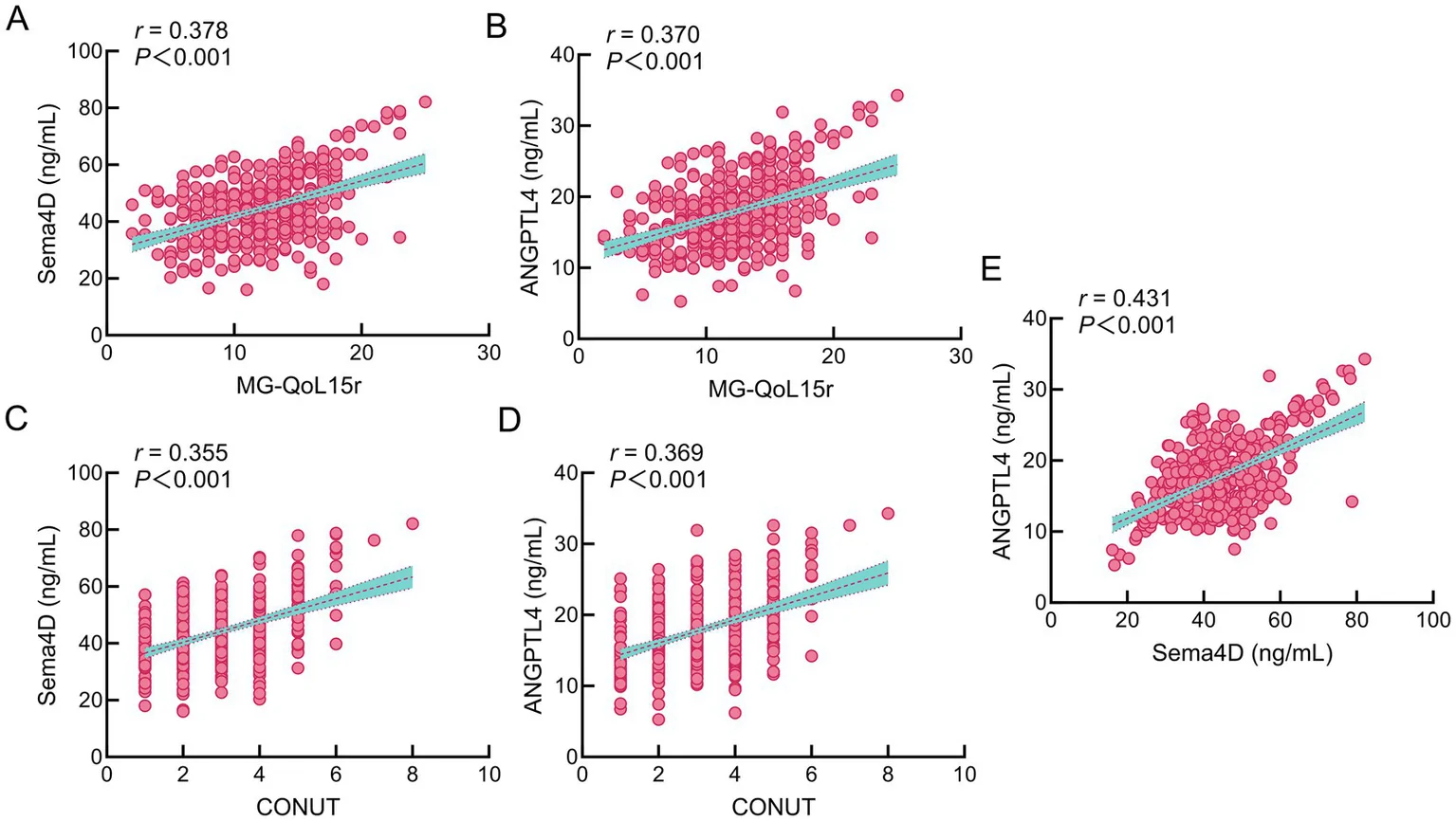
Correlation analyses of serum Sema4D and ANGPTL4 levels with MG-QoL15r and CONUT scores. **(A)** Correlation between Sema4D and the MG-QoL15r score; **(B)** correlation between ANGPTL4 and the MG-QoL15r score; **(C)** correlation between Sema4D and the CONUT score; **(D)** correlation between ANGPTL4 and the CONUT score; **(E)** correlation between Sema4D and ANGPTL4.

### 3.5 Predictive value of serum Sema4D and ANGPTL4 for poor prognosis in patients with MG

ROC curve analysis showed that serum Sema4D and ANGPTL4 individually yielded AUCs of 0.770 and 0.759, respectively, for predicting poor prognosis in patients with MG, whereas their combination yielded an AUC of 0.841, with sensitivity and overall predictive performance superior to either marker alone (Table 4, Figure 5).

**Table 4:** ROC curve analysis of serum Sema4D and ANGPTL4, alone and in combination, for predicting poor prognosis in MG patients.

Variable	Cutoff	AUC	Sensitivity (%)	Specificity (%)	*p*	95% CI
Sema4D	48.24 ng/mL	0.770	69.51	74.35	<0.001	0.707–0.832
ANGPTL4	20.35 ng/mL	0.759	57.32	81.82	<0.001	0.698–0.820
Sema4D + ANGPTL4	—	0.841	78.05	79.22	<0.001	0.790–0.893

**Figure 5:**
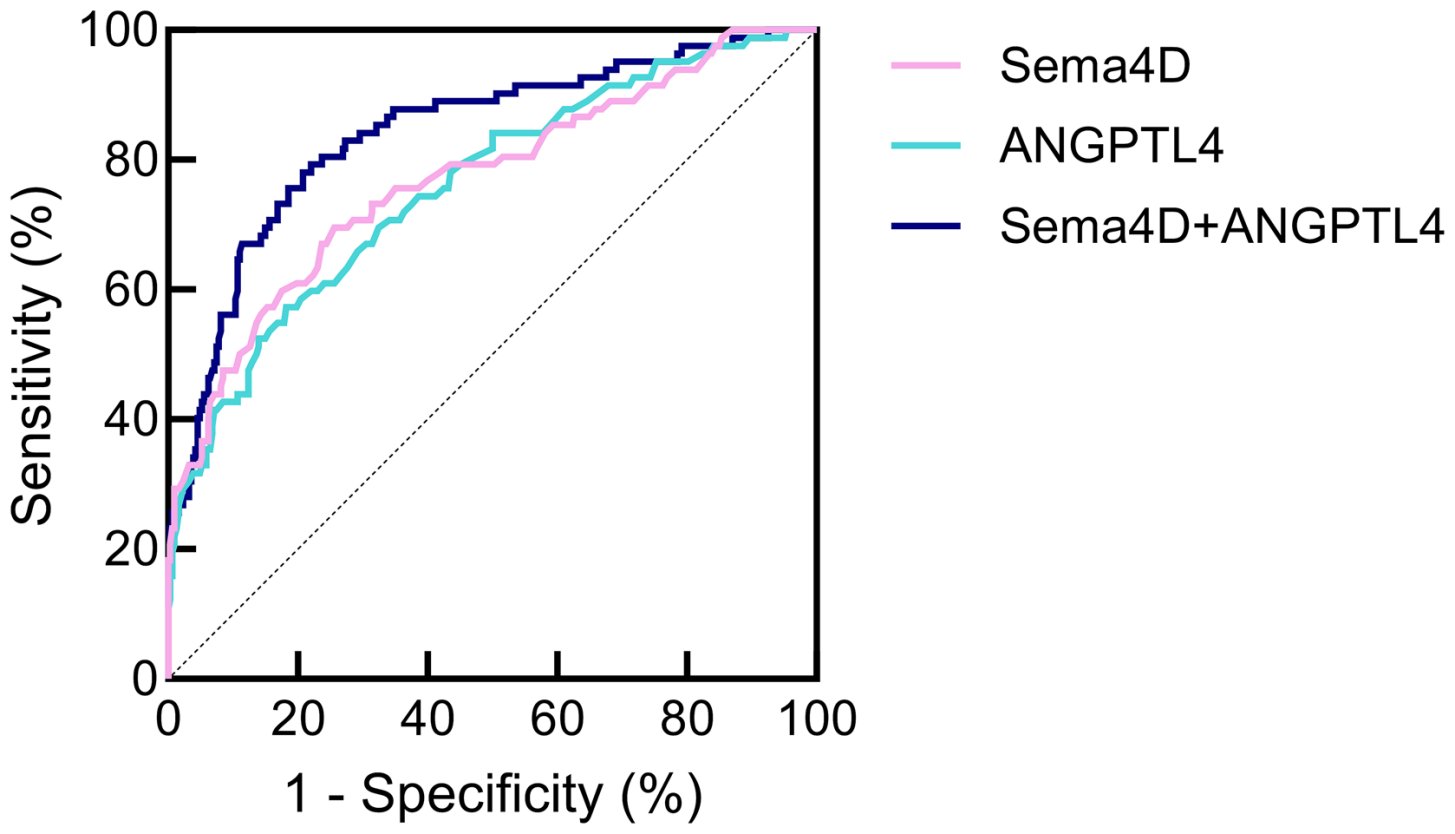
ROC curves of serum Sema4D and ANGPTL4, alone and in combination, for predicting poor prognosis in patients with MG.

### 3.6 Multivariable logistic regression analysis of factors associated with poor prognosis in patients with MG

Prognosis served as the dependent variable (good prognosis = 0, poor prognosis = 1). MGFA classification (type IV-V = 1, type I–III = 0), baseline dysphagia (present = 1, absent = 0), and thymoma (present = 1, absent = 0) were included as binary variables, while the MG-QoL15r score, CONUT score, and serum Sema4D and ANGPTL4 levels were included as continuous variables. Multicollinearity diagnostics indicated that the variance inflation factor (VIF) was below 2 for all predictors, suggesting no multicollinearity. The results showed that MGFA class IV-V, baseline dysphagia, thymoma, elevated MG-QoL15r score, elevated CONUT score, and increased serum levels of Sema4D and ANGPTL4 were all independent risk factors for poor prognosis (all *p* < 0.05) (Table 5).

**Table 5:** Multivariable logistic regression analysis of factors associated with poor prognosis in patients with MG.

Variable	*β*	S.E.	Wald	*p*	OR	95% CI	VIF
MGFA class IV-V	0.820	0.338	5.892	0.015	2.269	1.171–4.398	1.194
Baseline dysphagia	0.791	0.324	5.973	0.015	2.207	1.170–4.163	1.015
Thymoma	1.099	0.401	7.516	0.006	3.002	1.368–6.586	1.012
MG-QoL15r score	0.111	0.046	5.925	0.015	1.118	1.022–1.223	1.328
CONUT score	0.278	0.134	4.307	0.038	1.320	1.016–1.717	1.352
Sema4D	0.050	0.019	7.268	0.007	1.051	1.014–1.090	1.701
ANGPTL4	0.081	0.039	4.231	0.040	1.085	1.004–1.172	1.724

### 3.7 Incremental prognostic value of serum Sema4D and ANGPTL4 in MG patients

To assess the incremental prognostic value of Sema4D and ANGPTL4 beyond established clinical variables, we constructed a clinical baseline model (model 1) comprising only the MGFA class IV-V, dysphagia, thymoma, MG-QoL15r score, and CONUT score. The AUC of model 1 for predicting poor prognosis was 0.874 (95% CI: 0.835–0.913). When Sema4D (model 2) and ANGPTL4 (model 3) were added separately, the AUC increased to 0.890 (95% CI: 0.854–0.927) and 0.893 (95% CI: 0.859–0.928), respectively. DeLong tests showed that the AUC of each model differed significantly from that of the baseline model (*Z* = −1.986, *p* = 0.047; *Z* = −3.143, *p* = 0.002; Figures 6A,B). Incorporation of both Sema4D and ANGPTL4 (model 4) further increased the AUC to 0.907 (95% CI: 0.875–0.939), which was also significantly different from that of the baseline model (*Z* = −3.698, *p* = 0.0002; Figure 6C).

**Figure 6:**
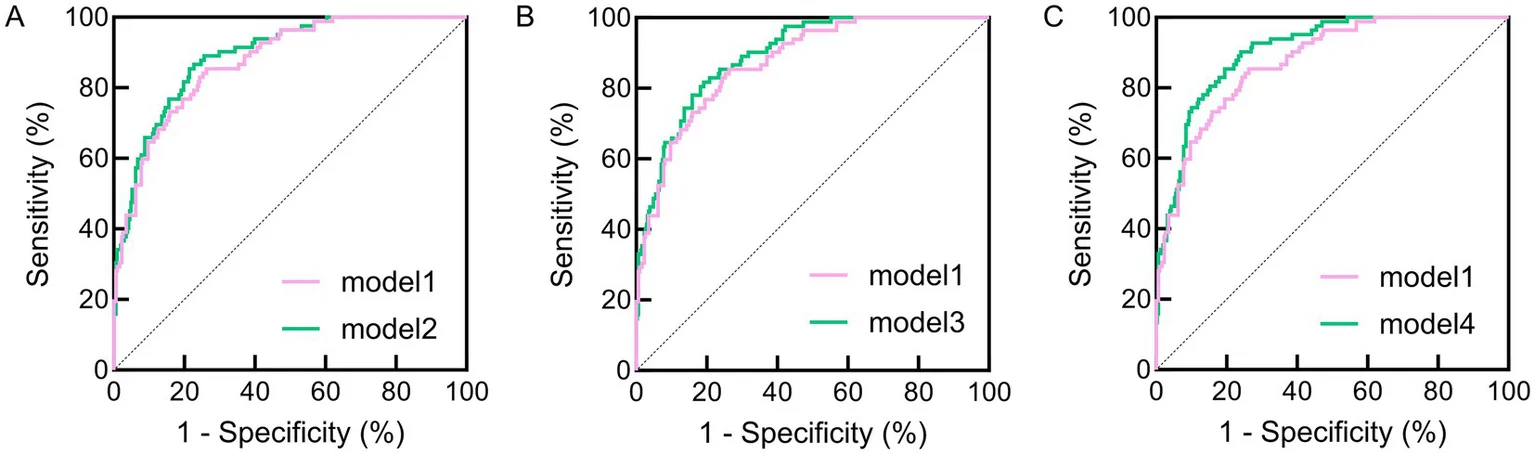
Comparison of ROC curves for the clinical baseline model and the models incorporating Sema4D and ANGPTL4. **(A)** Comparison of ROC curves between the clinical baseline model (model 1) and the model incorporating Sema4D (model 2); **(B)** comparison of ROC curves between the clinical baseline model (model 1) and the model incorporating ANGPTL4 (model 3); **(C)** comparison of ROC curves between the clinical baseline model (model 1) and the model incorporating both Sema4D and ANGPTL4 (model 4).

## 4 Discussion

In the present study, we observed elevated serum levels of Sema4D and ANGPTL4 in patients with MG. These levels were correlated with clinical severity as defined by the MGFA classification, quality-of-life impairment as assessed by the MG-QoL15r, and nutritional status as evaluated by the CONUT score. Both biomarkers were elevated in the poor-prognosis group, and their combined assessment demonstrated prognostic value. These findings suggest that changes in Sema4D and ANGPTL4 levels are associated with disease activity and severity in MG; however, the specific pathways through which they act and their potential clinical utility require further investigation.

The present study showed that MGFA class IV-V, baseline dysphagia, concomitant thymoma, higher MG-QoL15r scores, and higher CONUT scores were independent risk factors for poor prognosis in patients with MG. As the MGFA classification directly reflects disease severity, patients in higher classes often have more extensive muscle involvement and greater disease activity and are therefore more likely to experience adverse outcomes (20). Dysphagia suggests more severe bulbar involvement and may increase the risks of aspiration, infection, and malnutrition, whereas a higher CONUT score indicates poorer nutritional status, which may thereby affect prognosis (21). Because patients with MG often have dysphagia resulting from bulbar involvement and are at high risk of malnutrition, the present study used the CONUT score to assess nutritional status. However, nutritional status may also be influenced by disease severity, and the CONUT score may partly reflect MG severity rather than serve as a fully independent prognostic factor. Although the multivariable analysis was adjusted for MGFA classification and dysphagia, residual confounding may persist, and the independent effect of nutritional status should be verified in future mediation analyses. Patients with thymoma-associated MG typically exhibit more complex immune abnormalities and more severe clinical manifestations and have a relatively poor prognosis (22). A higher MG-QoL15r score reflects greater disease burden and functional impairment and is closely associated with disease progression (23). In this study, age did not differ significantly between the good-prognosis and poor-prognosis groups, suggesting that age may not be a key determinant of prognosis in MG; this finding may be related to the limited sample size and relatively narrow age range.

Sema4D is a transmembrane glycoprotein with functions in both axon guidance and immunomodulation and is widely expressed on the surface of immune cells, including T cells, B cells, and monocytes. In recent years, the roles of Sema4D and its related signaling pathways in immune diseases have attracted increasing attention, and Sema4D has shown potential as a diagnostic biomarker and therapeutic target (24, 25). ANGPTL4 is a member of the angiopoietin-like protein family and is involved in the regulation of inflammatory responses, vascular homeostasis, and energy metabolism (26). Recent studies have shown that both proteins are closely associated with the development and progression of various autoimmune diseases. Levels of Sema4D are elevated in rheumatoid arthritis, multiple sclerosis, psoriasis, and other autoimmune diseases, and this protein participates in immune-inflammatory processes, including T-cell activation, regulation of B-cell function, and inflammatory cell recruitment (27–29). Levels of ANGPTL4 are significantly elevated in autoimmune diseases, including rheumatoid arthritis and systemic lupus erythematosus, and this protein is involved in lipid metabolism and pathological processes such as synovial inflammation and renal injury (30–32). In addition, ANGPTL4 gene transcripts were significantly upregulated in extraocular muscle fibroblasts from patients with refractory ocular MG after exposure to serum from patients with MG (33). These studies suggest that both Sema4D and ANGPTL4 may serve as important biomarkers reflecting autoimmune abnormalities and inflammatory burden. However, studies examining the expression of Sema4D and ANGPTL4 in MG and their associations with disease severity and prognosis remain limited.

This study showed that serum levels of both Sema4D and ANGPTL4 were significantly higher in patients with MG than in healthy controls and that both biomarkers were present at higher levels in the poor-prognosis group than in the good-prognosis group. In addition, patients with MGFA class IV-V disease had markedly higher serum levels of both biomarkers than those with MGFA class I–III disease. Both biomarkers were positively correlated with MG-QoL15r and CONUT scores. Serum levels of both biomarkers were also higher in patients with thymoma than in those without thymoma, suggesting that thymoma-associated immune dysregulation may partly contribute to their upregulation. However, each biomarker remained independently associated with poor prognosis in the multivariable analysis after adjustment for thymoma, indicating that their elevation was not solely attributable to thymoma. These findings suggest that increased Sema4D and ANGPTL4 levels may be associated with greater clinical severity as reflected by the MGFA classification, reduced quality of life, and worsening nutritional status. Given that MG is fundamentally an autoimmune disorder of the neuromuscular junction, persistent immune activation and inflammatory responses during disease progression may promote increased release and expression of Sema4D and ANGPTL4. With increasing disease burden and declining functional status, their levels may rise further, consistent with their observed correlations with disease severity and impaired quality of life. Furthermore, combined assessment of Sema4D and ANGPTL4 significantly improved prognostic discrimination when added to established clinical variables, suggesting additional predictive value beyond conventional risk factors. Notably, this observational study could not elucidate the specific biological mechanisms underlying the involvement of Sema4D and ANGPTL4 in MG progression; these hypotheses require validation in further basic research.

Of note, serum Sema4D levels were positively correlated with serum ANGPTL4 levels, suggesting a tendency toward concordant changes in these two biomarkers during the development and progression of MG. However, direct mechanistic evidence supporting this correlation is currently lacking. On the one hand, Sema4D and ANGPTL4 are each closely associated with immune and inflammatory responses and may reflect immune activation and inflammatory burden. On the other hand, both are associated with inflammatory cell activation and tissue injury, suggesting that they may be regulated by similar pathological factors. In addition, mechanistic indicators were not collected in this study, precluding determination of whether a direct molecular interaction or causal relationship exists between them. Therefore, this correlation should be regarded as an observational finding that requires further validation.

In summary, elevated serum Sema4D and ANGPTL4 levels were associated with greater disease severity and poor prognosis in patients with MG, and their combined assessment may help evaluate prognostic risk. However, this study has several limitations. First, this was a single-center prospective study with a relatively small sample and a follow-up period of only 12 months; therefore, the findings require further validation in multicenter studies with larger cohorts and longer follow-up. Second, only patients with anti-AChR antibody–positive MG were enrolled; therefore, the conclusions may not be generalizable to patients with anti-MuSK antibody–positive, anti-LRP4 antibody–positive, or seronegative MG. Disease-specific quantitative measures, such as MG-ADL, QMG, and MGC scores, were also not systematically collected. Third, immunotherapy regimens during follow-up were individualized by clinicians according to disease status, resulting in treatment heterogeneity. The CONUT score may also have been influenced by dysphagia and other bulbar symptoms, partly reflecting disease severity rather than serving as a fully independent prognostic factor. Furthermore, the observational design precluded establishment of causal relationships between the two biomarkers and poor prognosis, and serial serum measurements during treatment were not performed; therefore, their potential as markers of treatment response remains unclear. Future studies should validate their prognostic value in broader populations and explore their utility in guiding individualized treatment and assessing therapeutic efficacy.

## Data Availability

The raw data supporting the conclusions of this article will be made available by the authors on reasonable request.
